# Bee Bread: Physicochemical Characterization and Phenolic Content Extraction Optimization

**DOI:** 10.3390/foods9101358

**Published:** 2020-09-24

**Authors:** Florina Dranca, Florin Ursachi, Mircea Oroian

**Affiliations:** Faculty of Food Engineering, Stefan cel Mare University of Suceava, 720229 Suceava, Romania; florina.dranca@usm.ro (F.D.); florin.ursachi@fia.usv.ro (F.U.)

**Keywords:** bee bread, polyphenols, extraction, fatty acids, sugars

## Abstract

Beebread or ambrosia is a unique product for humans and bees, which is the result of lactic fermentation on pollen in honeycombs. Bee bread is a rich source of nutrients (proteins, vitamins) and polyphenols (such as flavonoids, flavonols, phenolic acids). This study aimed to characterize bee bread in terms of physicochemical properties: pH, free acidity, glucose, fructose, sucrose, raffinose and melesitose content, total phenolic content (TPC), total flavones content (TFC), fatty acids and individual phenolics (gallic acid, protocatechiuc acid, *p*-hydroxybenzoic acid, caffeic acid, vanillic acid, chlorogenic acid, *p*-coumaric acid, rosmarinic acid, myricetin, luteolin, quercetin and kaempferol). The main phenolic compound identified in the bee bread was kaempferol, followed by myricetin and luteolin. The TPC, TFC and extraction yield were optimized in function of ultrasonic amplitude, temperature and time and the suitable conditions for achieving the maximum level were 87.20% amplitude of ultrasonic treatment, 64.70 °C and 23.10 min, respectively for reaching 146.2 mg GAE/L of TPC, 1231.5 mg QE/g of TFC and a 5.72% extraction yield. The most abundant fatty acids were C18:3 (all-*cis*-9,12,15) octadeca-6,9,15-trienoic acid, followed by C16:1 (9*Z*)-hexadec-9-enoic acid, C21:0 heneicosanoic acid and C18:2 (all-*cis*-9,12) (9Z,12Z)-octadeca-9,12-dienoic acid, respectively.

## 1. Introduction

Bee bread or ambrosia is a unique product for humans and bees, and is formed from pollen, honey and secretions of bees’ salivary glands [1,2] which are packed by the bees into the honeycomb and covered with wax and honey. During the preservation, the content is submitted to lactic fermentation caused by bacteria and yeast in the bee nest medium [3], thus resulting in bee bread which has a caramel color [4], a tangy taste which is given by its citrus, flowers or other fruit flavors [5]. Bee bread is the main food for the beehive, and particularly for larvae and young worker bees that produce royal jelly [3]. It can be considered an important source of nutrients and bioactive compounds such as proteins, vitamins (C, B, K, P and E), and polyphenols (flavonoids, phenolic acids) which are influenced by the source of pollen and different factors that influence the growth of the pollen plant (geographic origin, soil, climate) [6,7,8]. The studies carried out on volatile profile components showed a high concentration of unsaturated aliphatic acids, amino acids, terpene and terpene derivates, carbohydrates (e.g., glucose, fructose), and nitriles [9,10,11,12,13,14,15,16]. Bee bread is a rich source of polyphenols which includekaempherol, *p*-coumaric acid and isorhamnetin [10], apigenin, chrysin, naringenin, caffeic, ferulic and gallic acids, and quercetin [11,15]. Otham et al. [14] reported five phenolic compounds, as follows: Caffeic and ferulic acids, kaempferol, apigenin, and isorhamnetin in stingless bee bread. Kaplan et al. [17] determined 37 fatty acidsin bee bread, of which(9Z,12Z)-octadeca-9,12-dienoic, (9Z,12Z,15Z)-octadeca-9,12,15-trienoic, (Z)-octadec-9-enoic, (Z)-icos-11-enoic, hexadecanoic and octadecanoic acids were found in the highest concentrations, while Human and Nicolson [18] quantified only 18 fatty acids in bee bread originating from an indigenous South African bee plant. Bee bread was reported to have antioxidant activity due to the presence of α-tocopherol, phenolic compounds [11,19,20], antimicrobial activity against some pathogenic bacteria (*Pseudomonas aeruginosa*, *Salmonella enterica*, *Staphylococcus aureus*, *Escherichia coli* and *Bacillus cereus*) [21,22], antitumor activity against different tumor cell lines [11,15], antihypertensive activity [20] and neuroprotective activity [23].

Ultrasonic waves are low frequency, high energy sound waves that are used for their propagation into the extraction solvents which generates alternate phases of compression and rarefaction that cause acoustic cavitation [24]. Acoustic cavitation induces sonochemical effects which are considered the driving force. In brief, acoustic cavitation refers to the formation of cavitation bubbles, their growth and subsequent collapse [25]. The mass transfer rate during the extraction of bioactive compounds is influenced by the cavitation processes during which bubbles are formed; the bubbles formed collapse on plant material surfaces and explode. The explosion increases the pressure on the cell structure leading to its disruption and the targeted compounds from the matrix pass into the solvent [26,27,28,29]. Ultrasonic waves have been used in the last years for the extraction of different compounds such as pectin from apple pomace [30], anthocyanins and phenolic compounds from eggplant peel [31], polyphenols from old tea leaves [32], polyphenol from hemp, flax and canola seed cakes [26], flavonols from pollen [33], and in all the cases, an increase of extraction efficiency was reported.

Box–Behnken design (BBD) is a second-order design with three levels, which was developed by Box and Behnken. Compared to central composite design, the BBD is much easier to realize because it requires less factor levels. BBD was used for the optimization of different processes such as the extraction of phenolics from eggplant peel [31], pectin from apple pomace [30], phenolic compounds from coffee waste [34], pectin from mango peel [35], phenolics from bee pollen [33].

The present study aimed to analyze bee bread based on the following properties: pH, free acidity, palynological analysis, glucose, fructose, sucrose, raffinose and melesitose, total phenolic content (TPC), total flavones content (TFC), fatty acids and phenolic acids (gallic acid, *p*-coumaric acid, protocatechiuc acid, *p*-hydroxybenzoic acid, caffeic acid, vanillic acid, chlorogenic acid, rosmarinic acid), flavonoids (myricetin, luteolin) and flavonolos (quercetin and kaempferol) and to optimize the extraction of biological compounds by means of a Box–Behnken design using three parameters (ultrasonic amplitude, temperature and time). To the best of our knowledge, there is no other study in the literature regarding the optimization of the extraction process of bioactive compounds from bee bread.

## 2. Materials and Methods

### 2.1. Materials

Bee bread was purchased in Iasi County, Romania. The sample was harvested in the spring of 2020. The sample was stored at −20 °C prior to any analysis. The bee bread was defatted using *n*-hexane (the oil was used for fatty acids characterization, while the defatted bee bread was used for bioactive compounds analysis). Methanol, Folin-Ciocalteau reagent, ethanol, AlCl_3_, sodium carbonate, ethyl ether, rosmarinic acid, *p*-coumaric acid, chlorogenic acid, vanillic acid, caffeic acid, *p*-hydroxybenzoic acid, protocatechiuc acid, gallic acid, kaempferol, quercetin luteolin and myricetin, fructose, glucose, sucrose, melesitose and raffinose were purchased from Sigma–Aldrich (Taufkirchen, Germany). FAMEs mix was purchased from Restek (Lisses, France).

### 2.2. Methods

#### Ultrasonic Extraction Procedure

For the extractions, 0.3 g of bee bread was mixed with 30 mL of ethanol 96%, according to the parameters presented in Table 1. The ultrasonic procedure was conducted in a Ti-H-15 ultrasonic bath (Elma, Singen, Germany) at 45 kHz and maximum power of 100 W. The coded values are established according to Box–Behnken design where −1 represents the minimum level of the independent variable range, 0 represents the mean level of the independent variable range and 1 represents the maximum level of the independent variable range.

### 2.3. Physicochemical Characterization

#### 2.3.1. pH and Free Acidity

For the analysis, 2 g of the bee bread sample were mixed with 5 mL of mili-Q water and after homogenization, the solution was titrated against NaOH (0.05 M) according to the method described by Costa et al. [36]. The pH range of the method was 2–12, while the free acidity method was considered completed when the pH of the solution reached 8.5.

#### 2.3.2. Nutritional Value

The Association of Official Agricultural Chemists (AOAC) International procedures were used for the determination of total fat (AOAC 920.85), total protein (AOAC 978.04) and ash (AOAC 920.85 [37]. Total carbohydrates of the sample were determined by the difference method and energetic contribution was calculated by to the following equation [38]:

(1) Energy (kcal)=4×(g protein+g carbohydrates)+9×(g fat)

Organic acids were determined using a method described by Pauliuc et al. [39] using a Schimadzu High Performance Liquid Chromatograph HPLC instrument (Kyoto, Japan) with diode-array detector DAD. A Phenomenex Kinetex^®^ 5 μm C18 100 Å HPLC Column 250 × 4.6 mm was used for the separation of the organic acids (gluconic acid, formic acid, butyric acid, propionic acid, lactic acid and acetic acid, respectively).

Free sugars, 1 g of bee bread was mixed with 40 mL of 80% ethanol (*v*/*v*) and extracted for 30 min at 80 °C using Pinela et al.’s method. The resulting extract was centrifuged at 4000 rpm for 10 min. The supernatant was defatted three times with 10 mL of ethyl ether. The supernatant was concentrated and transferred into a 5 mL flask and filled with water. Prior to the HPLC determination the solution was filtered with a 0.2 μm Whatman nylon filter [40]. The separation of the free sugars (fructose, glucose, sucrose, melesitose and raffinose) was realized on the Schimadzu HPLC instrument (Kyoto, Japan) with RID (refractive index detector). A Phenomenex Luna^®^ Omega 3 μm SUGAR 100 Å HPLC Column 150 × 4.6 mm was used for the separation.

#### 2.3.3. Extraction Yield

To determine the extraction yield, 2 mL of bee bread extract were evaporated in a vacuum oven to constant weight [41]. The extraction yield was calculated as:

(2) $$\mathrm{Extraction}\;\mathrm{yield}=\;\frac{m_{\mathrm{de}}}{m_{e}}\cdot 100,\;\frac{g}{100g}\;\mathrm{bee}\;\mathrm{bread}$$

where m_de_ is the weight of the dried extract (g), and m_we_ is the weight of extract (g).

#### 2.3.4. Total Phenolic Content (TPC)

The TPC was determined using the method proposed by Escriche and Juan-Borras [41]. In a glass tube, 100 µL of extract was mixed with 1900 µL MiliQ of water and 100 µL of Folin–Ciocalteau reagent. After 2 min,800 µL of 5% sodium carbonate was added. The solution was thermostated at 40 °C for 20 min and cooled down in an ice bath to stop the reaction. The TPC concentration was expressed as mg gallic acid equivalent/L (mg GAE/L).

#### 2.3.5. Total Flavone Content (TFC)

The analysis of total flavone content was made according to the method previously described by Popova et al. [42]. In a volumetric flask, 2 mL of extract was mixed with 20 mL of methanol and 1 mL of 5% AlCl_3_ (prepared in methanol). The reaction lasted for 30 min and the solution’s absorbance was measured at 425 nm. The extract concentration was expressed as mg quercetin equivalent/L (mg QE/L).

#### 2.3.6. Phenolic Compounds Separation and Detection

The ethanolic extract was analyzed for the individual phenolics (rosmarinic acid, *p*-coumaric acid, chlorogenic acid, vanillic acid, caffeic acid, *p*-hydroxybenzoic acid, protocatechiuc acid, gallic acid, kaempferol, quercetin luteolin and myricetin) using a Schimadzu HPLC instrument (Kyoto, Japan) with DAD using a method described by Oroian et al. (Oroian, Ursachi, & Dranca, 2020a, 2020b). A Zorbax SP-C18 column, with 150 mm length, 4.6 mm i.d. and 5 μm-diameter particle was used for the separation.

#### 2.3.7. Fatty Acids Determination

Fatty acid derivation was made by the following procedure: 0.1 g of oil from bee bread (extracted with *n*-hexane for 48 h at room temperature) was mixed with 1 mL of m-hexane and 1 mL of 15% BF_3_ in methanol. The solution was heated at 60 °C for 15 min in a water bath. The solution was then cooled at room temperature and mixed with 5 mL of saturated NaCl solution and centrifuged for 5 min at 3000 rpm. The supernatant was filtered through a 0.45 µm nylon filter. The fatty acids separation and quantification was made on a GC-MS instrument from Shimadzu (GC MS-QP 2010 Plus, Shimadzu, Kyoto, Japan), based on the method described by Oroian et al. [33]. The composition of fatty acids was determined using calibration curves for each fatty acid using FAME’s mixed (Restek, Lisses, France) expressed as µg/g bee bread and as relative level (%) in fatty acids composition.

#### 2.3.8. FT-IR

The Fourier-transform infrared spectroscopy FT-IR spectra of the ehtanolic extract was realized using a Nicolet i-20 spectrophotometer (Thermo Scientific, Karlsruhe, Dieselstraße, Germany) in Attenuated total reflectance ATR mode, with the range of the wave number from 4000 to 650 cm^−1^ at a resolution of 4 cm^−1^. All the analyses were made on triplicate by placing the sample directly on the ATR crystal.

#### 2.3.9. Experimental Design and Statistical Analysis

A three-level three-factorBox–Behnken design was used for the modelling of the bee bread bioactive compounds extraction process (Table 1). The parameter modelling (TPC, TFC and extraction yield) was done using Design Expert 16 software (trial version, StatEase, Minneapolis, MN, USA) with a second-order polynomial response as:

(3) $$y=b_{0}+\overset{n}{\underset{i=1}{\sum }}\left(b_{i}x_{i}\right)+\overset{n}{\underset{i=1}{\sum }}\left(b_{\mathrm{ii}}x_{\mathrm{ii}}^{2}\right)+\overset{n}{\underset{\mathrm{ij}}{\sum }}\left(b_{\mathrm{ij}}x_{i}x_{j}\right)$$

## 3. Results and Discussions

### 3.1. Physicochemical Characterization of Bee Bread

In Table 2, the physicochemical parameters of the bee bread analyzed are presented; as it can be observed, the highest concentration was determined in the case of carbohydrates, followed by proteins. The fat content and ash content were found in a lower concentration than carbohydrates and proteins. The bee bread energy (412.07 Kcal/100 g) is higher than the value reported by Bakour et al. [8] in Moroccan bee bread. The fat content of bee bread reported in the literature ranged differently, as follows: 1.90% in Moroccan bee bread [8], 5.93% to 11.55% in clover, cotton, chestnut, citrus, and sunflower bee bread from Turkey [17], and 4.51% to 4.92% in Colombian bee bread [7].

The free sugars composition is presented in Table 2, and it can be observed that the main sugar was fructose (19.73%), followed by glucose (8.82%), and small amounts of melesitose and raffinose were also observed. The sucrose concentration was under detection limit; this may be due to the fact that during fermentation, sucrose is used by bacteria as a source of oxygen to produce different metabolites [43].

The analyzed bee bread had a 97.9 g/kg organic acids content, and the gluconic acid was the organic acid that was present in the highest concentration (79.2 g/kg), followed by acetic acid (10.4 g/kg) and formic acid (6.75 g/kg). To the best of our knowledge, there is no other study in the literature which has reported the composition of bee bread in organic acids. Bakour et al. [8] determined the total composition of organic acids expressed as oxalic acid content. Due to the fact that bee bread contains a high percentage of pollen, its organic acids composition can be compared to that of pollen. Kalaycioglu [44] analyzed anzer pollens and chestnut pollens and observed that they contain a high concentration of gluconic acid (5.9–32 g/kg) and lactic acid (0.72–1.2 g/kg). Recent studies confirm that organic acids can be considered as a new generation of growth promoters instead of antibiotics, and that bee bread can be considered a potent antibiotic due to the high concentration of organic acids [44].

### 3.2. Extraction Optimization

The ultrasonic amplitude had a positive influence on the extraction of TFC (*p* < 0.01), while for TPC and extraction yield, this process parameter had no significant influence (*p* > 0.05). Beebread is considered an important source of flavonoids [8] and the increase of the cavitation effects of ultrasound may increase the extractability of these compounds [33]. In the scientific literature, there are some papers reporting on the positive outcome of the use of ultrasounds for the extraction of different bioactive compounds from *Nepheliumlappaceum* [45], crude pollen [33], eggplant peel [46], grape seeds [47], as well as tea [32] and *Acer truncatum* leaves [48]. The increase of the extraction of TFC is due to the cavitation process generated by the ultrasound waves (24–50 kHz) which involve the cavitation bubbles formation and collapse in the media.

In Table 3, the influence of the temperature of extraction on the yield, TPC and TFC from bee bread is presented. For all three parameters (extraction yield, TPC and TFC) the influence of temperature was significant (*p* < 0.01); the temperature increased the solubility of the bioactive compounds into ethanol as a result of an increased cell permeability to the solvent [33] and of the disruption of the structure of cellular matrix [49]. The viscosity of the solvent decreased as the temperature increased and thus the penetration of the solvent into the cell structure improved [50,51]. Similar results were reported for the extraction yield from crude pollen [33], bioactive compounds from aromatic plants [52], wild sage [53], TPC from eggplant peel [31,46] and propolis [54]. The increase of temperature from 35 °C to 65°C improved the extraction yield with 52.4%, TPC extraction with 43.95% and TFC extraction with 27.8%, respectively.

The influence of extraction time on the yield and TPC and TFC extraction from bee bread is presented in Table 3; time significantly influenced all three parameters studied (*p* < 0.01). The extraction parameters increased with the increasing of the temperature, as follows: Extraction yield with 50.1%, TPC with 36.1% and TFC with 53.8%, respectively. The increase of extraction time led to a higher extraction efficiency because the solvent can penetrate the cell membrane for a longer time which increases the transfer rate from the cell into the solvent [55,56].

To study and optimize the ultrasound-assisted extraction process of bioactive compounds from bee bread, a response surface method - RSM model was implemented via a three-level three-factor Box–Behnken design; the optimization was made based on extraction yield, TPC and TFC. In Table 4, the statistical parameters (R^2^, adj-R^2^, coefficient of variance, F-value and *p*-value) of the second-order polynomial response surface model used for the experimental modeling of TPC, TFC and extraction yield are presented.

The regression coefficients of all three output parameters were higher than 0.9507, which confirmed the usefulness of the model. The F-values of the models (10.71 for extraction yield, 12.39 for TPC and 23.90 for TFC) were higher, while the *p*-values (<0.01) were lower, confirming that the model is appropriate to the experimental data. The models have a good accuracy and consistency (CV% is 8.80 for extraction yield, 12.136 for TPC and 16.577 for TFC).

The optimum extraction conditions were chosen based on a desirability function, and are 87.20% amplitude of ultrasonic treatment, 64.70 °C and 23.10 min, respectively. The extraction under optimum conditions reached 146.2 mg GAE/L of TPC, 1231.5 mg QE/g of TFC and a 5.72 g/100 g bee bread extraction yield.

### 3.3. Phenolic Compounds Composition

The phenolic acids, flavonoids and flavonols were determined in the extract with the highest total phenolic concentration (amplitude 60%, temperature 65 °C, time 25 min). As Table 5 shows, the highest concentration was determined for kaempferol (31.25 mg/L), followed by myricetin (3.15 mg/L), luteolin (1.17 mg/L) and rosmarinic acid (0.23 mg/L). In the extract, gallic acid, protocatechuic acid, *p*-hydroxybenzoic acid, vanillic acid and chlorogenic acid were not determined.

There are several studies in the scientific literature related to the phenolic composition of bee bread. Markiewicz-Zukowska et al. [11] analyzed the phenolic composition of Polish bee bread and reported trace amounts of kaempferol and apigenin. Baltrusaityte et al. [57] reported *p*-coumaric acid, kaempferol, apigenin and chrysin as main phenolics in beebread from Lithuania. In Ukrainian beebread, naringenin, kaempferol, apigenin, isorhamnetin and quercetin were determined [10]. Urcan et al. demonstrated that there is a high correlation between the flora pollen, bee pollen and bee bread, and that there is no correlation between soil/climate and phenolic composition [16]. The phenolic composition of bee bread is not affected by the transformations that happen during the fermentation. Bakour et al. [8] reported thirteen compounds, represented mainly by flavonols glycoside derivatives, especially quercetin (quercetin-*O*-hexosyl-*O*-hexoside, quercetin-*O*-hexosyl-*O*-hexoside, quercetin-*O*-pentosyl-hexoside and quercetin 3-*O*-rutinoside), kaempferol (kaempferol-*O*-hexosyl-*O*-rutinoside and kaempferol-3-*O*-rutinoside), isorhamnetin (isorhamnetin-*O*-hexosyl-*O*-rutinoside, isorhamnetin-*O*-pentosyl-hexoside, isorhamnetin-*O*-pentosyl-hexoside, isorhamnetin-*O*-rhamnoside-hexoside and isorhamnetin-3-*O*-rutinoside, respectively), and methylherbacetrin (methylherbacetrin-*O*-dihexoside and methylherbacetrin-3-*O*-rutinoside) derivatives.

### 3.4. Fatty Acid Composition

The fatty acids found in bee bread oil were quantified using Gas chromatograph coupled with mass spectrometer GC-MS. A total number of 37 fatty acids were determined by this analysis method and are presented in Table 6. Bakour et al. [8] quantified 25 fatty acidsin Moroccan bee bread, Kaplan et al. [17] quantified 37 fatty acids in Turkish bee bread, while Čeksterytė et al. [58] reported 39 fatty acids in Lithuanian bee bread. Human and Nicolson [18] reported 18 fatty acids in the composition of bee bread originating from South Africa, which included long-chain, saturated and monounsaturated fatty acids.

The total concentration of fatty acids in bee bread oil was 3110.85 µgg^−1^. The most abundant fatty acids were C18:3 (all-*cis*-9,12,15) Octadeca-6,9,15-trienoic acid, followed by C16:1 (9*Z*)-hexadec-9-enoic acid and C21:0 Heneicosanoic acid and C18:2 (all-*cis*-9,12) (9Z,12Z)-octadeca-9,12-dienoic acid, respectively. The high level (45.54%) of polyunsaturated fatty acids (PUFAs) was mainly determined by the high content of the oil fraction of bee bread in α-linoleic acid (C18:3 (all-*cis*-9,12,15) Octadeca-6,9,15-trienoic acid, 30.68%); this level exceeded the values obtained by Bakour et al. [8] in the case of Moroccan bee bread. Some fatty acids (e.g., oleic and palmitic acids) are important for the nutrition of honey bees, while other fatty acids (e.g., myristic, linoleic and linolenic acids) play an important role in inhibiting the growth of spore-forming bacteria and other microbes [59].

### 3.5. FT-IR Analysis

**Figure 1:**
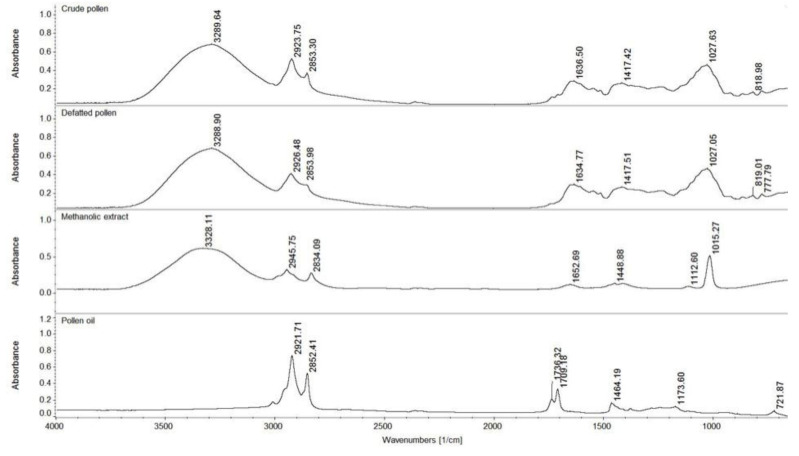
Fourier-transform infrared spectroscopy FT-IR spectra for bee bread, ethanolic extract and bee bread oil.

Figure 1 shows the FT-IR spectra for bee bread, bee bread extract (resulted from the extraction at an ultrasonic amplitude of 60%, temperature 65 °C, time 25 min) and bee bread oil recorded in the medium infrared region (MIR) of 4000–650 cm^−1^. As bee bread is a fermented mixture of plant pollen, honey, and secretions of the salivary glands of bees [3,4] its FT-IR spectra resembled that of crude pollen, which we have characterized in a previous study [33]. In the region 4000–2000 cm^−1^, bee bread shared similar absorption bands to pollen at 3286.25 cm^−1^, which corresponded to O–H stretching vibrations and was due to the presence of water [60], and also at 2916.78 cm^−1^ and 2849.01 cm^−1^, where the peaks were attributed to symmetric and asymmetric stretching of the C–H groups in carbohydrates (glucose, fructose, sucrose, arabinose) and lipids contained by the sample [13,61]. Other peaks related to the lipid fraction were observed at 1734.67 cm^−1^ and 1417.20 cm^−1^ and were assigned to C=O stretching in the ester bond and C–H deformation vibrations of lipids, respectively [61,62]. The broad band at 1636.05 cm^−1^, which was also attributed to O–H bending vibrations, overlapped the C=O stretching vibrations from amide I region (1640 cm^−1^), indicating protein composition [33,62]. The peak at 1541.06 cm^−1^ was also related to the protein fraction of bee bread and corresponded to N–H deformation and C–N stretching vibrations from amide II [61]. The large peak at 1030.19 cm^−1^ corresponded to C–O vibration in carbohydrates [60,62].

The FT-IR spectra of bee bread extract displayed the same stretching vibrations in the region 4000–2000 cm^−1^, attributed to the presence of water and lipids that were recorded for bee bread. Characteristic to the presence of lipids was also the C–H vibration at 1379.25 cm^−1^. The peaks at 1456.62 cm^−1^ and 879.52 cm^−1^ were due to stretching vibrations of the aromatic ring [63], while the peaks at 1087.16 cm^−1^ and 1044.74 cm^−1^ were attributed to C–OH bending and C–C elongation in alcohol groups [64]. In the case of the oil extracted from bee bread, the symmetric and asymmetric stretching modes of C–H groups (2916.38 cm^−1^ and 2848.61 cm^−1^) in alkyl chains, the C=O stretching in ester bond (1734.77 cm^−1^), the stretching of carboxylic group (1710.71 cm^−1^) and the C–H vibration (1375.49 cm^−1^) were all characteristic to lipids. The peaks at 1463.06 cm^−1^ and 1172.38 cm^−1^ were due to C–C vibrations of the aromatic ring, while the peak at 719.52 cm^−1^ indicated vibrations in C–OH groups [60].

## 4. Conclusions

Bee bread is an important source of different compounds (mainly phenolics) which present antioxidant, antimicrobial, antitumor and neuroprotective activity. The present paper presents a comprehensive characterization of bee bread from physicochemical point of view, phenolics and fatty acids composition. The main phenolic determined in the ethanoic extract was kaempferol, followed by myricetin and luteolin. Bee bread shared similar absorption bands to pollen at 3286.25 cm^−1^, which corresponded to O–H stretching vibrations and was due to the presence of water, and also at 2916.78 cm^−1^ and 2849.01 cm^−1^, where the peaks were attributed to symmetric and asymmetric stretching of the C–H groups in carbohydrates (glucose, fructose, sucrose, arabinose) and lipids contained inthe sample. Our study presents an optimized extraction process of phenolics from bee bread using ultrasound, being the first study in the literature regarding the optimization of the process using ultrasound at different amplitudes (20–100%), temperatures (35, 50 and 65 °C) and extraction times (5 min, 15 min and 25 min). The optimum extraction conditions were 87.20% amplitude of ultrasonic treatment, 64.70 °C and 23.10 min, respectively, and resulted in an extraction of 146.2 mg GAE/L of TPC and 1231.5 mg QE/g of TFC, and a 5.72 g/100 g bee bread extraction yield.

## Figures and Tables

**Table 1:** Actual and coded values of experimental design.

Independent Variables	Coded Values		
	−1	0	1
Ultrasonic amplitude (%)—X_1_	20%	60%	100%
Temperature (°C)—X_2_	35	50	65
Time (min)—X_3_	5	15	25

**Table 2:** Physicochemical parameters of bee bread.

Parameter		Value
pH		4.04
Free acidity (mEq/kg)		543.2
Ash content (%)		3.42
Fat content (%)		5.15
Protein content (%)		18.60
Carbohydrates (%)		72.83
Energy (kcal/100g)		412.07
Free sugars	Fructose (%)	19.73
	Glucose (%)	8.82
	Sucrose (%)	ND *
	Melezitose (%)	0.97
	Raffinosse (%)	0.96
Organic acids	Gluconic acid (g/kg)	79.2
	Formic acid (g/kg)	6.75
	Lactic acid (g/kg)	ND
	Acetic acid (g/kg)	10.4
	Succinic acid (g/kg)	ND
	Propionic acid (g/kg)	1.30
	Butyric acid (g/kg)	0.33

* ND—not detected.

**Table 3:** ANOVA of the influence of extraction parameters on total phenolic content (TPC), total flavonoid content (TFC) and extraction yield. Mean values and standard deviation.

Parameter	Ultrasonic Amplitude (%)			F-value	Temperature (°C)			F-Value	Time (min)			F-Value
	20	60	100		35	50	65		5	15	25	
Extraction yield (g/100 g bee bread)	3.75 (1.02) a	4.38 (0.98) a	4.19 (1.10) a	2.85 ns	3.25 (0.77) c	4.13 (0.87) b	5.03 (0.68) a	43.73 **	3.19 (0.86) c	4.38 (0.88) b	4.74 (0.67) a	35.82 **
TPC (mg GAE/L)	101.2 (21.4) a	112.8 (18.9) a	108.5 (26.8) a	2.05 ns	89.3 (9.7) c	108.3 (19.7) b	128.5 (10.9) a	59.27 **	89.1 (17.9) c	112.5 (18.3) b	121.3 (16.4) a	39.87 **
TFC (mg QE/L)	759.4 (138.9) c	840.3 (150.8) b	944.8 (266.6) a	31.75 **	745.4 (142.4) c	837.4 (198.6) b	963.9 (166.0) a	39.87 **	667.3 (128.2) c	846.5 (132.4) b	1026.1 (147.2) a	44.09 ***

GAE—gallic acid equivalent, QE—quercetin equivalent, ns—*p* > 0.05, **—*p* < 0.01, ***—*p* < 0.001, a–c Different letters in the same row indicate significant differences among samples (*p* < 0.05).

**Table 4:** ANOVA analysis of response surface method (RSM) model response for extraction yield, TPC and TFC from bee bread.

Source	DF	Extraction Yield (g/100 g Bee Bread)		TPC (mg GAE/L)		TFC (mg QE/L)	
		F-Value	*p*-Value	F-Value	*p*-Value	F-Value	*p*-Value
Model	9	12.93	0.0089	12.39	0.0064	23.90	0.0014
X_1_	1.00	2.85	0.1519	2.05	0.2112	31.75	0.0024
X_2_	1.00	43.73	0.0012	59.27	0.0006	44.09	0.0012
X_3_	1.00	35.82	0.0019	39.87	0.0015	118.75	0.0001
X_12_	1.00	2.47	0.1772	0.88	0.3912	14.17	0.0131
X_13_	1.00	0.91	0.3831	0.28	0.6170	4.08	0.0994
X_23_	1.00	0.01	0.9561	0.01	0.9561	1.40	0.2907
$X_{1}^{2}$	1.00	5.42	0.0674	5.32	0.0691	0.30	0.6067
$X_{2}^{2}$	1.00	0.01	0.9255	0.01	0.8795	0.58	0.4811
$X_{3}^{2}$	1.00	5.73	0.0621	4.53	0.0865	0.01	0.9241
R^2^		0.9507		0.9571		0.9773	
Adj-R^2^		0.8619		0.8799		0.9364	
CV%		8.80		6.62		5.50	
Adeq.Pre		10.190		12.136		16.577	

GAE—gallic acid equivalent. QE—quercetin equivalent. R^2^—regression coefficient, Adj-R^2^—regression coefficient adjusted, CV—coefficient of variance.

**Table 5:** Phenolic profile determined for the extract obtained under optimum extraction conditions.

Compound	Concentration (mg/L Extract)
Gallic acid	ND
Protocatechuic acid	ND
*p*-Hydroxybenzoic acid	ND
Vanillic acid	ND
Caffeic acid	0.10
Chlorogenic acid	ND
*p*-Coumaric acid	0.11
Rosmarinic acid	0.23
Myricetin	3.15
Luteolin	1.17
Quercetin	0.06
Kaempferol	31.25

ND—not detected.

**Table 6:** Fatty acids composition of bee bread oil.

Fatty Acid	Concentration (µg/g Bee Bread)	Relative Level (%)
C4:0 Butanoic acid	39.47	1.27
C6:0 Hexanoic acid	12.82	0.41
C8:0 Octanoic acid	5.96	0.19
C10:0 Decanoic acid	2.45	0.08
C11:0 Undecanoic acid	35.05	1.13
C12:0 Dodecanoic acid	9.42	0.30
C13:0 Tridecanoic acid	3.33	0.11
C14:0 Tetradecanoic acid	7.71	0.25
C14:1 (cis-9) (Z)-tetradec-9-enoic acid	14.93	0.48
C15:0 Pentadecanoic acid	21.11	0.68
C15:1 (cis-10) (Z)-pentadec-10-enoic	18.26	0.59
C16:0 Hexadecanoic acid	7.35	0.24
C16:1 (9Z)-hexadec-9-enoic acid	656.37	21.10
C17:0 Heptadecanoic acid	14.30	0.46
C17:1 cis-10-heptadecenoic	17.89	0.57
C18:0 Octadecanoic acid	3.51	0.11
C18:1 (E)-octadec-9-enoic	112.87	3.63
C18:1 (Z)-octadec-9-enoic	140.80	4.53
C18:2 (all-trans-9,12) Octadeca-9,12-dienoic acid	1.16	0.04
C18:2 (all-cis-9,12) (9Z,12Z)-octadeca-9,12-dienoic acid	319.89	10.28
C18:3 (all-cis-6,9,12) Octadeca-6,9,12-trienoic acid	7.98	0.26
C18:3 (all-cis-9,12,15) Octadeca-6,9,15-trienoic acid	954.51	30.68
C20:0 Icosanoic acid	37.61	1.21
C20:1 (cis-11) (Z)-icos-11-enoic acid	11.32	0.36
C20:2 (all-cis-11,14,) Icosa-11,14-dienoic acid	8.50	0.27
C20:3 (all-cis-8,11,14) (8Z,11Z,14Z)-icosa-8,11,14-trienoic acid	21.19	0.68
C20:3 (all-cis-11,14,17) (11Z,14Z,17Z)-icosa-11,14,17-trienoic acid	10.41	0.33
C20:4 (all-cis-5,8,11,14) (5Z,8Z,11Z,14Z)-icosa-5,8,11,14-tetraenoic acid	0.93	0.03
C20:5 (all-cis-5,8,11,14,17) (5Z,8Z,11Z,14Z,17Z)-icosa-5,8,11,14,17-pentaenoic	72.34	2.33
C21:0 Heneicosanoic acid	369.04	11.86
C22:0 Docosanoic acid	0.35	0.01
C22:1 (cis-13) (Z)-docos-13-enoic	0.88	0.03
C22:2 (all-cis-13,16) Docosa-13,16-dienoic	12.55	0.40
C22:6 (all-cis-4,7,10,13,16,19) Docosa-4,7,10,13,16,19-hexaenoic	7.10	0.23
C23:0 Tricosanoic acid	135.75	4.36
C24:0 Tetracosanoic acid	14.45	0.46
C24:1 (cis-15) (Z)-tetracos-15-enoic	1.29	0.04
Total	3110.85	
Saturated fatty acids (%)	23.13%	
Unsaturated fatty acids (%)	76.87%	
Mono-unsaturated fatty acids (%)	31.33%	
Poli-unsaturated fatty acids (%)	45.54%	
